# Efficacy and safety of a combination of emotional freedom technique with acupuncture versus acupuncture alone to treat psychiatric symptoms in Parkinson’s disease: A protocol for a randomized, assessor-blind, parallel-group clinical trial

**DOI:** 10.1097/MD.0000000000033714

**Published:** 2023-05-26

**Authors:** Dong-Hoon Kang, Ju-Yeon Kim, Yang-Chun Park, Ho-Ryong Yoo, In Chul Jung

**Affiliations:** a Department of Oriental Neuropsychiatry, College of Korean Medicine, Daejeon University, Daejeon, Republic of Korea; b Department of Internal Medicine, College of Korean Medicine, Daejeon University, Daejeon, Republic of Korea.

**Keywords:** emotional freedom technique, acupuncture, Parkinson’s disease, psychiatric symptoms

## Abstract

Parkinson’s disease (PD) is one of the most prevalent neurodegenerative diseases in world. As some psychiatric symptoms degrade the quality of life of patients with PD, a novel alternative non-pharmacological treatment is required. Acupuncture appears to be an effective and safe treatment for PD. The emotional freedom technique (EFT) is a type of psychological therapy that alleviates psychiatric symptoms by stimulating acupoints. In this study, we will compare the efficacy and safety of a combination of the EFT and acupuncture and acupuncture alone.

**Methods:**

This study is a randomized, assessor-blind, parallel-group clinical trial. Eighty participants will be equally divided into experimental and control groups. Each participant will receive a total of 24 interventions over 12 weeks. The experimental group will receive EFT combined with acupuncture and the control group will receive acupuncture alone. The primary outcome is the change in the Beck Depression Inventory score from baseline to 12 weeks, and the secondary outcomes include change in the following variables: Beck Depression Inventory, Parkinson’s disease sleep scale, State-Trait Anxiety Inventory, the Korean version of the Fatigue, Resistance, Ambulation, Illnesses, and Loss of weight questionnaire scale, and unified Parkinson’s disease rating scale III and exercises.

**Discussion:**

Acupuncture is a safe and effective treatment for motor and nonmotor symptoms in PD, and EFT appears to be safe and effective for a variety of psychiatric symptoms. In this study, we will investigate the potential of EFT combined with acupuncture to improve psychiatric symptoms in PD.

## 1. Introduction

Parkinson’s disease (PD) is a widespread disease with its prevalence predicted to be 100 to 300 per 100,000 people.^[1]^ As the incidence of PD increases with age, the number of patients with PD is expected to increase significantly due to the general aging of the population.^[2]^ Therefore, the prevention, treatment, and management of PD are becoming increasingly important. Approximately 35% of patients with PD have depression, and their activities of daily living and cognitive function decrease faster than those without depression.^[3,4]^

Acupuncture is a clinically effective and safe treatment for motor and nonmotor symptoms such as psychiatric disorders, sleep problems, and gastrointestinal symptoms in patients with PD.^[5,6]^

The emotional freedom technique (EFT) is a type of psychological therapy that assesses targeted psychological issues.^[7]^ It alleviates psychological problems by stimulating acupoints without acupuncture.^[8]^ The EFT has been proven to effectively improve depression, anger, and anxiety in a variety of populations^[8–10]^ and improve a variety of diseases such as sleep disorders, constipation, allergies, and blood pressure.^[7]^

In South Korea, the increase in social and health care costs due to PD is emerging as an important social problem.^[11,12]^ Therefore, the demand for drugs or health technologies that treat the psychiatric symptoms of PD is continuously increasing.

The main pathogenesis of PD is the degeneration of the dopaminergic area, which results in not only motor symptoms such as bradykinesia and resting tremors, but also psychiatric symptoms such as dementia, cognitive decline, depression, and sleep disorders.^[13]^ In addition, motor disability in PD can lead to severe negative social consequences and easily result in depression.^[14]^ These psychiatric symptoms affect the quality of life of patients and caregivers.^[15]^ Therefore, various approaches, including continuous management and treatment, are required for PD against accompanying nonmotor symptoms.^[16]^ Since the psychiatric symptoms of PD are closely related to changes in motor and nonmotor symptoms, the main conventional treatment for PD is dopamine supplementation, and anti-depressants can be used to alleviate depression and anxiety symptoms.^[17]^ However, dopamine supplementation may worsen the psychiatric symptoms of PD and anti-depressants may be related to the deterioration of motor symptoms.^[15]^ Therefore, studies about safer complementary non-pharmacological treatment options are required.

In this study, we will compare the efficacy, safety, and economic efficiency of EFT combined with acupuncture and acupuncture alone for the psychiatric symptoms of PD.

## 2. Methods/design

### 2.1. Study design

This study is a randomized, assessor-blind, parallel-group clinical trial. This clinical trial will be conducted at Daejeon Korean Medicine Hospital of Daejeon University. This protocol (version 1.6, February 2022) complies with the Recommendations for Interventional Trials (SPIRIT) Standard Protocol guidelines. All items from the World Health Organization Trial Registration Data set are described in Table S1, Supplemental Digital Content, http://links.lww.com/MD/I936.

### 2.2. Study procedure

Before conducting this clinical trial, the investigator will explain the “Informed consent form” to the participants and receive written consent from the participants for the clinical trial based on their free will after ensuring that they have sufficiently understood what is required of them. The subjects will be given a screening code after obtaining their consent.

# DJ-S-ZZZ [DJ: Daejeon Korean Medicine Hospital of Daejeon University, S: initial screening, ZZZ: Serial number (001, 002–)] (ex. DJ-S-012: 12th applicant for screening of Daejeon Korean Medicine Hospital of Daejeon University).

The eligible subjects who meet the inclusion and exclusion criteria will be selected based on a demographic information survey, vital signs (blood pressure, pulse rate, and body temperature), physical examination, medical history (including chief complaint, onset, motif, past history, smoking, and alcohol ingestion), Hoehn & Yahr scale, 15-item geriatric depression scale (GDS-15), Korean Mini-Mental State Examination (MMSE-K), chest X-ray, electrocardiography (EKG), and laboratory tests at screening. The included subjects will be assigned an identification code.

# DJ-E-ZZZ [DJ: Daejeon Korean Medicine Hospital of Daejeon University, E: initial enrollment, ZZZ: Serial number (001, 002–)] (ex. DJ-E-012: 15th registered subjects of Daejeon Korean Medicine Hospital of Daejeon University).

Screening tests should be conducted within 7 days of visit 1 (baseline visit), and visit 25 should be conducted within 18 weeks (±3 days). At visit 1, the investigator will check the results of the screening test (including chest X-ray and laboratory tests) and changes in medical history and concomitant drugs between the screening and visit 1. The subjects will be randomized and allocated to experimental or control groups and provided with an identification code. Before the intervention, subjects will complete the Beck Depression Inventory (BDI), Parkinson’s disease sleep scale (PDSS), State-Trait Anxiety Inventory (STAI), the Korean version of the Fatigue, Resistance, Ambulation, Illnesses, and Loss of weight questionnaire scale (K-FRAIL), Unified Parkinson’s Disease Rating Scale III (UPDRS III), exercise measurement, fNIRS (device name: NS1-H20AM), efficacy assessment (EQ-5D, EQ-VAS), and cost analysis. The experimental group will be treated with EFT and acupuncture, while the control group will be treated with acupuncture only. At visits 2 to 24, the investigator will check for adverse effects and changes in medical history and concomitant drugs, and subjects will receive EFT combined with acupuncture or acupuncture according to the corresponding intervention schedule at each visit. Tests and assessments will be conducted according to the following schedule: visit 12 (BDI, PDSS, STAI, K-FRAIL, UPDRS III, efficacy assessment, cost analysis, exercise measurements); visit 24 (BDI, PDSS, STAI, K-FRAIL, UPDRS III, fNIRS, efficacy assessment, cost analysis, exercise measurements); and visit 25 (follow-up study; BDI, PDSS, STAI, K-FRAIL, UPDRS III, efficacy assessment, cost analysis, and exercise measurements). The detailed schedules are listed in Table 1.

**Table 1:** Time schedule.

Period	Screening	Treatment					Follow up
Week	-1	0–6 W ± 1 D			7 12 W ± 1 D		18 W ± 3 D
Visit	Screening	1	2–11	12	13–23	24	25
Consent	●						
Hoehn & Yahr	●						
GDS-15	●						
MMSE-K	●						
Demographic information	●						
EKG	●						
Chest X-ray	●						
Lab test	●				●		
Pregnancy test	●				●		
Medical history	●	●	●	●	●	●	●
Vital signs	●	●	●	●	●	●	●
Eligibility screen	●						
Randomization		●					
Height, B/W		●		●		●	●
Check concomitant drugs		●	●	●	●	●	●
Check Adverse effects			●	●	●	●	●
Acupuncture		●	●	●	●	●	
EFT		●	◎	◎	◎	◎	
BDI		●		●		●	●
PDSS		●		●		●	●
STAI		●		●		●	●
K-FRAIL		●		●		●	●
UPDRS Ⅲ		●		●		●	●
fNIRS		●				●	
Efficacy assessment		●		●		●	●
Cost analysis		●		●		●	●
Exercise measurements		●		●		●	●
Compliance measurements			●	●	●	●	
Education of visit schedule		●	●	●	●	●	

### 2.3. Clinical assessments

In this study, the clinical assessments are as follows: vital signs (blood pressure, pulse rate, and body temperature); laboratory tests (complete blood cell count: hemoglobin, hematocrit, red blood cell count, white blood cell count, platelet count, erythrocyte sedimentation rate; blood chemistry test: total protein, albumin, aspartate transaminase, alanine transaminase, gamma-glutamyl transpeptidase, total bilirubin, blood urea nitrogen, creatinine, glucose, Na+, K+, Cl−, free thyroxine, thyroid-stimulating hormone; urine analysis: specific gravity, pH, erythrocyte, leukocyte, nitrite, protein, glucose, ketone, urobilinogen, bilirubin, microscopy); pregnancy test (urine human chorionic gonadotropin, only for fertile women, to ensure that the results are negative); EKG (12 leads electrocardiography); chest X-ray; GDS-15; Hoehn & Yahr Scale; BDI-II; PDSS; STAI; K-FRAIL; UPDRS III; EQ-5D; EQ-VAS; cost analysis; MMSE-K.

### 2.4. Study populations

#### 2.4.1. Inclusion criteria.

1. Aged 45 to 85 years
2. Diagnosed with PD
3. Hoehn and Yahr Scale score of 1 to 3
4. The 15-item Geriatric Depression Scale (GDS-15) score of over 6
5. MMSE-K score of over 24
6. Participants who voluntarily decided to participate and signed the consent form

#### 2.4.2. Exclusion criteria.

1. Patients with dementia, Huntington’s disease, or hydrocephalus
2. Patients with gait disturbance due to cerebral vascular accidents, brain tumors, or other cerebral diseases
3. Participants who have taken or changed the dosage of antiparkinson medication, materials, or medications that may affect depression (e.g., anti-anxiolytic drugs, anti-depressive drugs, anti-psychotic drugs, steroids, female hormones, L-dopa, digitalis, bromide, cyclosporin, disulfiram, isoniazid, yohimbine), in the previous 2 weeks
4. Patients who cannot participate in the trial due to lab test results (e.g., over 2 times the upper reference limit of aspartate transaminase, alanine transaminase, total bilirubin, or creatinine)
5. Seriously unstable medical conditions (the investigator will decide based on vital signs, EKG, and chest radiograph by the standard operating procedure. E.g., active tuberculosis on chest radiograph or signs of ventricular fibrillation or myocardial infarctions on EKG)
6. Participants who have been treated with Korean medical treatment related to PD in the past 2 weeks, since that treatment may affect this trial or its safety.
7. Pregnant or lactating women
8. Participants who are thought to be not appropriate to participate in this trial by the investigator.

#### 2.4.3. Sample size.

The statistical hypothesis test of the evaluation variable is two-sided and the significance level will be set at 5%. The type 2 error(β) will be set to 0.2, and the power will be maintained at 80%. The ratio of the experimental and control groups should be 1:1. We expect the BDI reduction and standard deviation of the acupuncture group to be 2.8 and 7.8, respectively, and the BDI reduction of the experimental group to be 7.8, based on previous studies conducted with a design similar to this study.^[18,19]^ Therefore, the required sample size for each group is expected to be “2*6.92(1.96 + 0.84)2/(7.8 − 2.8)2 ≃ 30/group.” In addition, considering the dropout rate of 25%, we have decided to register 40 participants per group, with a total of 80 participants.

#### 2.4.4. Randomization, blinding.

The people in charge of other assignments (or independent statisticians), who are not involved in conducting and evaluating this clinical trial, will generate a random assignment list in a specific and reproducible manner. A block randomization method will be used for randomized allocation. The random assignment table will be kept sealed and managed separately by the research manager in the presence of the participants. Each group will be randomly assigned with the chance of each individual being selected using the statistical program SAS® version 9.4 (SAS Institute. Inc., Cary, NC). The investigators and assessors will be separated so that the investigators will not know what kind of treatment the subjects have received. It is not possible to maintain investigator blindness.

### 2.5. Interventions

The EFT combined with the acupuncture group (experimental group) will receive EFT once a week for 12 weeks (a total of 12 times) and acupuncture treatment twice a week for 12 weeks (a total of 24 times). In the experimental group, acupuncture will be performed after the EFT. Acupuncture treatment will be performed on a total of 15 acupoints: GV20, GV24, EX-HN3, as well as bilaterally at LI11, PC6, HT7, ST36, SP6, and KI3” for better clarity. The acupuncture alone group (control group) will receive acupuncture treatment twice a week for 12 weeks (a total of 24 times). Acupuncture treatment will be the same in both the experimental and control groups. The STRICTA results of acupuncture treatment are described in Table 2.

**Table 2:** STRICTA of acupuncture treatment.

STRICTA list		Description
1)Acupuncture rationale	a) Style of acupuncture	Meridian of Traditional Korean Medicine
	b, c) Reasoning for treatment provided, based on historical context, literature sources, and/or consensus methods, with reference where appropriate	Textbook of the meridian, Korean medical clinical practice guidelines for Parkinson’s disease, depression, literature review of acupuncture for Parkinson and depression.
2)Needling details	a) Names (or location if no standard name)	GV20, GV24, EX-HN3, LI11, PC6, HT7, ST36, SP6, KI3
	b) Number of needle insertions per subject per session	15
	c) Depth of insertions, based on a specified unit of measurement, or on a particular tissue level	Vertical insertion, 5–30 mm
	d) Response sought	None
	e) Needle stimulation	Manual
	f) Needle retention time	30 min
	g) Needle type	0.25 mm × 30 mm, Dongbang medical, stainless steel
3)Treatment regimen	a) Number of treatment sessions	24
	b) Frequency and duration of treatment sessions	twice a week for 12 weeks (24 times in total)
4)Co-intervention	a) Details of other interventions administered to the acupuncture group	Emotional Freedom Technique (EFT) (only in the experimental group)
5)Practitioner background	a) Description of participating acupuncturists	a specialist of neuropsychiatry or internal medicine of traditional Korean medicine, or a resident who is being educated by the specialist of neuropsychiatry or internal medicine of traditional Korean medicine, and have clinical experience in neuropsychiatry or internal medicine of traditional Korean medicine for over 1 year.
	b) Years in acupuncture practice	over 1 year.
	c) Qualification or professional affiliation	a specialist of neuropsychiatry or internal medicine of traditional Korean medicine, or a resident who is being educated by the specialist of neuropsychiatry or internal medicine of traditional Korean medicine, and has clinical experience for over 1 year.

EFT will be performed by a specialist in neuropsychiatry or internal medicine of traditional Korean medicine, or a resident who has been educated by a specialist in neuropsychiatry or internal medicine of traditional Korean medicine and has clinical experience in neuropsychiatry or internal medicine of traditional Korean medicine for over 1 year.

We will follow the EFT protocol suggested in the EFT manual (2008).^[20]^ It consists of 6 stages: goal setting and confirming the problem, setup, sequence, the 9 Gamut sequence, sequence, and EFT reframing. We modified the protocol and constructed it in 6 stages as described in Table 3.

**Table 3:** EFT protocol.

1)Goal setting and symptom identification	Decide a psychological or somatic symptom to resolve, and score present distress between 0 to 10.
2)Preparation	Repeat the following words while tapping or massaging the points on the hands and chest. (“Even though I feel uncomfortable about my ___, I deeply and completely accept myself.”)
3)Basic tapping	While repeating simple words that can remind the uncomfortable situations, tap each meridian point 7 times.
4)Brain tuning	While tapping TE3, close and open the eyes, and then move the pupils towards the lower right side, lower left side, turn clockwise, and then counterclockwise. Sing a single line of a simple song and count the number (1–5).
5)Basic tapping	Repeat 3) Basic tapping
6)Reevaluation and additional tapping	Reevaluate the present distress. If it was not changed at all, identify other issues or distress by 1) step. If it was slightly reduced, repeat 2) -5).

### 2.6. Criteria for concomitant drugs

#### 2.6.1. Possible drugs.

If doses of antiparkinson drugs and psychiatric drugs (e.g., anti-depressants, anti-anxiety medication, tranquilizers, and sleeping pills) are consistent for 4 weeks before participating in the trial, it is permissible. Drugs for transient care of other diseases will be medicated after confirmation by the researcher. When concomitant drugs, including drugs for other diseases or adverse events, are administered, information about the drugs, including name, purpose, dose, and duration, will be recorded in a progress note.

#### 2.6.2. Prohibited drugs.

There are no specific combination-prohibited drugs, but the drugs that can affect results can be prohibited depending on the decision of researchers (e.g., starting with new medications, such as anti-depressants, anti-anxiety drugs, tranquilizers, corticosteroids, female hormones, L-dopa, digitalis, bromide, cyclosporin, disulfiram, isoniazid, and yohimbine, during the clinical trial.).

### 2.7. Adverse event report

The investigator will report any severe adverse events to the principal investigator (PI) regardless of whether they are related to the intervention during the trial. In addition, events associated with the intervention that are considered by the investigator to be of significant danger will be recorded in the progress note as severe adverse events. In this study, the following are considered as severe adverse events: death; life-threatening events; the need for hospital admission or to extend the admission period; events resulting in continuous or severe disability or impairment of function; events requiring treatment due to other critical medical situations.

When severe adverse events occur, the PI will notify the client (Daejeon Korean Medicine Hospital of Daejeon University) immediately and provide an additional report, including details, within 5 days. The trial should be stopped until further instructions are provided. The client should promptly report any severe or unexpected adverse events to other relevant investigators, the Institutional Review Board (IRB), and the Director of the Ministry of Food and Drug Safety.

### 2.8. Criteria for discontinuation or dropout

#### 2.8.1. Discontinuation.

In the case of an adverse event that can have an adverse effect on the safety of participants or can affect the clinical trial, the ongoing test should be discontinued, excluding the safety assessment. The PI and the client will discuss the safety of the intervention and decide whether to proceed with or discontinue the trial.

#### 2.8.2. Dropout.

Subjects will be dropped if they fail to complete the trial due to adverse events or other reasons. The investigator can stop the interventions and tests and drop the subjects from the trial or the subjects can voluntarily quit the trial. The subjects may be dropped in the following cases: severe adverse events occurred to the subjects; difficulty proceeding with the trial due to adverse events; detecting systemic disease that was not found in the pre-intervention examination; the subject or a legal representative of the subject wants to stop the trial; the subject resists the instructions of the investigator; the subject withdraws consent to participate in the trial; the subject is lost to follow-up; the subject is prescribed a treatment that can affect study result, without the direction or consent of the investigator during the trial period or follow-up period; progression of the trial is considered inappropriate by the investigator.

### 2.9. Compliance assessment

Compliance assessment will be performed as follows:


$$\begin{array}{l} Compliance\;\left(\;\%\;\right)\;=\frac{Number\;of\;performed\;interventions}{Number\;of\;planned\;interventions} \end{array}$$

Compliance (%) during the trial should be at least 75%. If the compliance (%) of the subjects is less than 75%, it is considered poor and is excluded from the per-protocol (PP) analysis.

### 2.10. Statistical analysis

Efficacy analysis will be evaluated primarily based on the full analysis set principle and secondarily according to the PP principle of the primary outcome. Missing values will be imputed appropriately according to why they occur. This method is expected to conservatively evaluate the test results by considering the tendency of PD to worsen over time. Statistical analysis will be performed using the two-sided test, and *P* < .5 will be considered statistically significant.

### 2.11. Efficacy assessment

#### 2.11.1. Primary outcome.

The primary outcome will be assessed based on the change in the BDI score from baseline to 12 weeks. The change in the BDI score between the 2 groups will be verified using an independent *t* test. We will use analysis of covariance if there is a significant difference in the baseline and multiple regression analysis if there is a significant difference in the other base variables. The interaction between time and intervention will be tested using repeated analysis of variance of the BDI scores at each time point.

#### 2.11.2. Secondary outcome.

The secondary outcome will be assessed by changes in the BDI score from baseline to 6 and 18 weeks, PDSS, STAI, K-FRAIL scale, UPDRS III, exercise measurements from baseline to 6, 12, and 18 weeks, and fNIRS from baseline to 12 weeks. We will use analysis of covariance or multiple regression analysis to correct baseline variables for the efficacy assessment of BDI, PDSS, STAI, K-FRAIL, UPDRS III, and fNIRS variables. The baseline score will be used as a covariate, in which case a PP analysis will be performed without any handling of missing values.

### 2.12. Safety assessment

Changes in laboratory test results before and after the trial will be clinically evaluated. All adverse events during the trial will be described in detail. Data will be collected through patient self-reporting and researcher observation. The frequency of adverse events related to interventions and not related to interventions will be recorded and presented as descriptive statistics. A list of adverse reactions, including frequency, definite occurrence time, occurrence rate, severity, and intervention casualty, will be presented, with graphs if necessary. If statistical analysis is required, the paired *t* test, McNemar test, analysis of variance), *t* test, chi-square test, and/or Fisher’s exact test will be performed according to the characteristics of the variables and the purpose of the statistical evaluation.

### 2.13. Economic efficiency assessment

An economic analysis will be performed to check the cost-effectiveness of the intervention in this study and the usual care. The primary economic endpoint will be assessed using the cost per quality-adjusted life-year. The estimation of quality of life for the quality-adjusted life-year calculation will use the quality of life derived EQ-5D as the main evaluation variable, and the calculation will use the area under the curve method.^[21]^ The costs will be calculated by combining the number of treatments and cost units. The secondary economic endpoint will be assessed using the cost per EQ-VAS score.

The economic analysis will be primarily evaluated based on the full analysis set principle and secondarily based on the PP principle to check the sensitivity to the missing value analysis. Missing values will be imputed properly based on why they occur in the same manner as in the efficacy analysis. The first analysis period is 18 weeks, and if an estimation of the subsequent period is required, the second analysis will be estimated by extrapolation through the regression model or performed by the decision modeling analysis. The cost unit will be settled by 2021 Korean currency.

In this study, the analytical perspective is a social perspective. In the baseline analysis, the representative values (such as average) of parameters will be used, and in the sensitivity analysis, probabilistic sensitivity analysis will be performed through the distribution and representative values of all possible estimation parameters.

The results will be presented in tables including the incremental cost-effectiveness ratio, cost-effectiveness plane, cost-effectiveness acceptability curve, and value of information analysis graph. The cost-effectiveness plane will include the confidence interval of the non-parametric methods. The cost-effectiveness acceptability curve will show the sensitivity of cost-effectiveness according to the national threshold. The value of the information analysis graph will estimate the value of the information about the included population.

The statistical analysis of this study will be conducted after verifying the normality of all continuous variables through the Shapiro–Wilk test, and the statistical significance level will be tested with a *P* value of .05. Stata 14 MP version and R program (4.0.2 version) will be used as analysis programs, and Treeage Pro 2016 will be used as an analysis program if extrapolation is performed through modeling.

### 2.14. Data management

The investigators will collect medical information and record it in each patient’s case report form (CRF). The data will be saved confidentially in accordance with the personal information protection policy of the National Institute for Korean Medicine Development (NIKOM). A copy of the documents related to this trial including patient informed consent, records of participants, and CRFs will be kept for 3 years in document storage.

### 2.15. Monitoring

The monitoring will be conducted by phone calls and visits. The monitoring staff will be composed of the A-CRO (Academic Contract Research Organization) of Daejeon Korean Medicine Hospital, who is independent of investigators and funders. The staff will periodically check and review the data storage and the progress of the trial. After a monitoring session, a follow-up letter will be sent in 2 days to the investigators. The monitoring report will be completed in 7 days, reviewed, and approved in 4 days.

### 2.16. Ethics and dissemination

It was approved by the Institutional Review Board at Daejeon University Daejeon Medical center (DJDSKH-21-BM-04) and registered via Clinical information Services (CRIS) on February 25, 2021 (identifier: KCT0005964). All participants will receive an explanation about this trial details. The written Informed Consent Form will be obtained from all participants prior to enrollment.

### 2.17. Study protocol modifications

When revising this protocol, the date, the details of the revision, and the reason for the revision must be reported to the IRB and approved by the IRB. The change of the study protocol will also be notified to participants.

## 3. Discussion

PD is mainly characterized by motor symptoms, but cognitive and psychiatric symptoms are also commonly observed. These psychiatric symptoms are often underestimated but have negative effects on the quality of life of patients with PD and their caregivers.^[22,23]^ Although medications, such as anti-depressants, appear to be effective for PD psychiatric symptoms,^[24,25]^ they are sometimes ineffective or do not meet treatment needs.^[26,27]^ Therefore, additional or novel treatments are required to manage the psychiatric symptoms of PD.

EFT is a type of psychological therapy that alleviates psychiatric symptoms. It can be considered as a cognitive therapy using meridian by tapping acupoints.^[28]^ Cognitive therapy has been shown to be effective for a wide range of psychiatric symptoms such as anxiety, depression, and insomnia in PD.^[29,30]^ Therefore, we expect EFT to be effective in alleviating psychiatric symptoms in patients with PD.

This randomized, assessor-blind, parallel-group clinical trial protocol is to evaluate the safety and efficacy of EFT combined with acupuncture compared to acupuncture alone for the psychiatric symptoms in PD. We expect EFT combined with acupuncture will be used as a novel treatment for psychiatric symptoms associated with PD.

## Author contributions

**Conceptualization:** Yang-Chun Park, Ho-Ryong Yoo, In Chul Jung.

**Funding acquisition:** Ho-Ryong Yoo, In Chul Jung.

**Methodology:** Dong-Hoon Kang, Ju-Yeon Kim, Yang-Chun Park, Ho-Ryong Yoo, In Chul Jung.

**Writing – original draft:** Dong-Hoon Kang.

**Writing – review & editing:** Dong-Hoon Kang, Ju-Yeon Kim, In Chul Jung.

## Supplementary Material
