# Supplementary material for: Efficacy and safety of a combination of emotional freedom technique with acupuncture versus acupuncture alone to treat psychiatric symptoms in Parkinson’s disease: A protocol for a randomized, assessor-blind, parallel-group clinical trial
Source: Medicine (Baltimore). 2023 May 26;102(21):e33714. doi: 10.1097/MD.0000000000033714 (PMC10219728; doi:10.1097/MD.0000000000033714)
Supplement: Supplementary file 1 [file medi-102-e33714-s001.pdf]

Data category	Information
Primary registry and trial identifying number	KCT0005964 (Clinical information Services in Korea)
Date of registration in primary registry	February 25, 2021
Secondary identifying numbers	N/A
Source(s) of monetary or material support	This work was supported by the Daejeon University Research Grant (2021) and a grant of the Traditional Korean Medicine R&D Project, Ministry of Health & Welfare, Republic of Korea (HF20C0174).
Primary sponsor	Ministry of Health & Welfare, Republic of Korea
Secondary sponsor	Daejeon University
Contact for public queries	Professor In Chul Jung<br>npjeong@dju.kr<br>Department of Oriental Neuropsychiatry, College of Korean Medicine, Daejeon University, Republic of Korea
Contact for scientific queries	Professor In Chul Jung<br>npjeong@dju.kr<br>Department of Oriental Neuropsychiatry, College of Korean Medicine, Daejeon University, Republic of Korea
Public title	The Efficacy and Safety of a Combination of Emotional Freedom Technique with Acupuncture versus Acupuncture Alone to Treat Psychiatric Symptoms in Parkinson's disease: A Randomized Clinical Trial: A protocol for a randomized, assessor-blind, parallel-group clinical trial
Scientific title	The Efficacy and Safety of a Combination of Emotional Freedom Technique with Acupuncture versus Acupuncture Alone to Treat Psychiatric Symptoms in Parkinson's disease: A Randomized Clinical Trial: A protocol for a randomized, assessor-blind, parallel-group clinical trial
Countries of recruitment	Republic of Korea

Health condition or problem(s) studied	Parkinson's disease
Intervention(s)	Intervention – Combination of Emotional freedom technique with acupuncture<br>Control – Acupuncture alone
Key inclusion and exclusion criteria	Inclusion criteria - Aged 45 to 85 years; Diagnosed with PD; Hoehn and Yahr Scale score of 1-3; The 15-item Geriatric Depression Scale (GDS-15) score of over 6; Mini-Mental State Examination-K (MMSE-K) score of over 24<br>Exclusion criteria - Patients with dementia, Huntington's disease, or hydrocephalus; Patients with gait disturbance due to cerebral vascular accidents, brain tumors, or other cerebral diseases; Participants who have taken or changed the dosage of antiparkinson medication, materials, or medications that may affect depression in the previous two weeks; Patients who cannot participate in the trial due to lab test results; Seriously unstable medical conditions 6) Participants who have been treated with Korean medical treatment related to PD in the past two weeks, since that treatment may affect this trial or its safety; Pregnant or lactating women
Study type	Interventional<br>Allocation: randomized<br>Masked: single-blind<br>Primary purpose: evaluation of efficacy, safety, and economic efficiency
Date of first enrollment	September 06, 2021
Target sample size	80
Recruitment status	Recruiting
Primary outcome(s)	BDI score (baseline, after 12 weeks)
Secondary outcome(s)	1. BDI score (baseline, after 6, 18 weeks)<br>2. STAI (baseline, after 6, 12, and 18 weeks)<br>3. K-FRAIL scale (baseline, after 6, 12, and 18 weeks)<br>4. UPDRS III (baseline, after 6, 12, and 18 weeks)<br>5. Exercise measurements (baseline, after 6, 12, and 18 weeks)

Supplementary Table 1. All items from the World Health Organization Trial Registration Data Set
